# Phylogeographic and SNPs Analyses of *Bemisia tabaci* B Mitotype Populations Reveal Only Two of Eight Haplotypes Are Invasive

**DOI:** 10.3390/biology10101048

**Published:** 2021-10-15

**Authors:** Jorge R. Paredes-Montero, Q. M. Imranul Haq, Amr A. Mohamed, Judith K. Brown

**Affiliations:** 1 School of Plant Sciences, University of Arizona, Tucson, AZ 85721, USA; jrparedes@email.arizona.edu; 2 Facultad de Ciencias de la Vida, Escuela Superior Politécnica del Litoral, ESPOL, Campus Gustavo Galindo Km 30.5 Vía Perimetral, P.O. Box 09-01-5863, Guayaquil 090902, Ecuador; 3 Department of Biological Sciences and Chemistry, College of Arts and Sciences, University of Nizwa, Nizwa 616, Oman; quaziimran@unizwa.edu.om; 4 Department of Entomology, Faculty of Science, Cairo University, Giza 12613, Egypt; mamr@sci.cu.edu.eg

**Keywords:** biogeography, climate niche, diversity, phylogeny, whitefly

## Abstract

**Simple Summary:**

The whitefly *Bemisia tabaci* taxon consists of an undefined number of morphologically identical genetic variants of which only a few, including the B, harbor invasive haplotypes. These haplotypes have potential to upsurge and become important pests and plant virus vectors in irrigated agroecosystems worldwide. In the 1980s, unprecedented outbreaks associated with the B variant were reported worldwide, however, the precise origin(s) of the invasive haplotypes has not been determined. In this study, available *B. tabaci* mitochondrial gene sequences were examined for patterns of conserved single nucleotide changes (SNPs). The whitefly sequence records represented North Africa-Middle Eastern habitats, the proposed B variant center of origin, and distant locales recently invaded by haplotype(s) of the B variant. Unexpectedly, the analysis revealed eight SNPs groups (haplotypes) demonstrating that the genetic architecture of the B mitoype is more complex than previously recognized. Also, the distribution patterns of the eight B haplotypes were tightly linked to well-defined eco-geographic regions, suggesting the different groups have diversified by geographic isolation. Contrary to claims that collectively, the B variant is invasive, only two of the eight haplotypic groups have established in geographical locations outside of their zone of endemism.

**Abstract:**

The *Bemisia tabaci* cryptic species contains 39 known mitotypes of which the B and Q are best recognized for having established outside their extant endemic range. In the 1980s, previously uncharacterized haplotype(s) of the B mitotype rapidly established in tropical and subtropical locales distant from their presumed center of origin, leading to displacement of several native mitotypes and extreme damage to crops and other vegetation particularly in irrigated agroecosystems. To trace the natural and evolutionary history of the invasive B haplotypes, a phylo-biogeographic study was undertaken. Patterns of single nucleotide polymorphisms (SNPs) and signatures potentially indicative of geographic isolation were investigated using a globally representative mitochondrial cytochrome oxidase I gene (mtCOI) sequence database. Eight haplotype groups within the North Africa-Middle East (NAFME) region were differentiated, NAFME 1–8. The NAFME 1–3 haplotypes were members of the same population that is associated with warm desert climate niches of the Arabian Peninsula and east coastal Africa-Ethiopia. The NAFME 4 and 5 haplotypes are endemic to warm and cold semi-arid niches delimited by the Irano-Turanian floristic region, itself harboring extensive biodiversity. Haplotypes 6 and 7 co-occurred in the Middle East along eastern Mediterranean Sea landmasses, while NAFME 8 was found to be endemic to Cyprus, Turkey, and desert micro-niches throughout Egypt and Israel. Contrary to claims that collectively, the B mitotype is invasive, NAFME 6 and 8 are the only haplotypes to have established in geographical locations outside of their zone of endemism.

## 1. Introduction

The whitefly *Bemisia tabaci* cryptic (or sibling) species group [1,2,3] refers to a mostly polyphagous complex of morphologically cryptic, genetically-distinguishable biological variants distributed worldwide in tropical and sub-tropical regions [4,5,6]. Previously, seven major phylogeographic clades have been resolved based on phylogenetic analysis of the partial mitochondrial cytochrome oxidase (mtCOI) gene sequence [1,7], collectively comprising 39 genetically divergent mitotypes. Five of these major phylogeographic clades have been corroborated in an analysis of 2184 single copy orthologous nuclear genes [2].

Since the first identification of *B. tabaci* as a pest of tobacco crops in Greece [8], it has been increasingly recognized as an agricultural pest worldwide due to damage caused by feeding and as the vector of plant virus pathogens [9,10,11]. Large-scale agricultural crop damage by *B. tabaci* as a sporadic pest has been documented since the 1930s, particularly in cotton-vegetable cropping systems in Egypt, India, Israel, Sudan, and in Central and South America [9,12,13]. In the Jordan Valley, *B. tabaci* was shown to complete up to 15 generations per year due to the favorable hot climate there, and 11 generations in the coastal areas [8]. During the mid-1970s, unprecedented outbreaks and crop damage have been reported in Israel and Egypt, while another major outbreak occurred in Israel in 1988 [9]. A decline in severity was achieved by the mid-1990s following regular use of pyrethroids and other new insecticides [14]. Among the mitotypes of historical importance are the well-known B and Q mitotypes [1,15,16,17,18,19,20,21], the A mitotype endemic to the American tropics and Caribbean Basin [9,22], the Asia I and II in the Indian subcontinent [23,24,25,26], and sub-Saharan Africa mitotypes [27,28,29].

The discovery in the United States of *B. tabaci* colonizing poinsettia [30,31,32] and *Brassica spp.* [33], both non-hosts of *B. tabaci* endemic to the Americas [31], prompted genetic studies to identify the origin. Studies to differentiate mitotypes mainly relied on polymorphic esterase patterns [10,31,34,35,36,37,38]. The esterase patterns revealed at least three variants, B, B1, and B2 patterns [34], and showed that the poinsettia variant shared the B pattern, which was most like esterase patterns for *B. tabaci* from Egypt and Israel, whereas the B1 and B2 electromorphs best matched *B. tabaci* endemic to Cyprus and Yemen, respectively [10,34]. The geographical origin and climate niches associated with all three ‘B esterase types’ were traced to their region of endemism in the North Africa-Middle East (NAFME) region [7,38,39]. By 1990–1991, the B mitotype had displaced the endemic A mitotype in Arizona and California cotton and vegetable cropping systems [33,39], resulting in estimated losses over a 10-year period from 200 to 500 million dollars in Arizona, California, Florida, and Texas [40]. Widespread introductions of B mitotypes occurred throughout the rest of the Americas and Caribbean Basin during the 1980s-1990s [41]. However, the natural and evolutionary history of the B haplotype(s) that became invasive remains unknown.

Because mitochondrial genes evolve more rapidly compared to their nuclear gene counterparts, base substitutions accumulate that are valuable for studying patterns of geographic variation among populations [42,43,44]. In *B. tabaci* mitochondrial substitutions have begun to saturate compared to the nuclear genome [2]. However, the phylogenetic breaks resolved by this marker highly correlate with a geographic segregation pattern, suggesting the microevolutionary changes evident are useful for assigning local endemism of *B. tabaci* mitotypes [1,7,26,45,46,47]. With this new information and ease of haplotyping *B. tabaci* by a ‘DNA barcoding’ approach, large datasets are increasingly useful for studying large-scale species assemblages and populations, worldwide.

The objective of this study was to investigate the patterns of intraspecific genetic variation for representative populations of the B mitotype collected from the presumed native niche ranges, and from locales where it has been reportedly introduced. To determine whether the genetic architecture of the B mitotype can be explained phylo-bio-geographically, the congruence between extant B mitotype ecological niches and geographical placement of mtCOI phylogenetic breaks was investigated. To accomplish this, a mtCOI sequence dataset was compiled from records available in the GenBank database and for additional samples available in the Brown lab collection, School of Plant Sciences, The University of Arizona (Tucson, AZ, USA). The mtCOI sequences were scrutinized for ‘signature SNPs’, enabling classification of unique haplotypes, and subjected to phylogenetic analysis. The SNPs haplotype results were overlayed with the hypothetical native niche range(s) for the B mitotype cryptic species, which were categorized as ecogeographical units based on global Koppen-Geiger climate classification [48] and hotspots of predicted endemism. 

## 2. Materials and Methods

### 2.1. The mtCOI Sequences

In total, 591 sequences were downloaded from the NCBI nucleotide database using the Entrez search and retrieval system EDirect [49]. The esearch function was used to search the database with the following queries, together or separate: ‘cytochrome oxidase I gene’, ‘COI’, ‘*Bemisia tabaci*’, ‘B biotype’, ‘B mitotype’, ‘MEAM 1′, and ‘Middle East Asia Minor’. The function efilter was used to select mtCOI sequences by length, which ranged from 650–1000 bp. The filtered nucleotide sequences in ‘fasta’ format were downloaded using the efetch function. Validated reference sequences (morphologically- and molecular sequence-verified) not yet available in the GenBank database were selected from the laboratory database (Brown Lab, UA, Tucson, AZ, USA). Forty mtCOI sequences from the Brown Laboratory collection were determined from archived field samples and added to the dataset. The mtCOI sequences were obtained using standard methods established in the Brown laboratory for PCR amplification, cloning, and Sanger sequencing of selected archived adult *B. tabaci*. The 3′-mtCOI gene fragment [46,50] was amplified with the primers, C1-J-2195 (5′-TTGATTTTTTGGTCATCCAGAAGT) and L2-N-3014 (5′-TCCAATGCACTAATCTGCCATATTA), with cycling parameters consisting of 30 cycles of 95 °C for 30 s, 52 °C for 30 s, and 72 °C for 1 min. Amplicons were ligated using a pGEM-T Easy system (Promega, Madison, WI, USA). Competent *E. coli* DH5α cells were transformed using ligated plasmids. Colony PCR amplification [51] was carried out using primers M13F (5’-TGTAAAACGACGGCCAGT) and M13R (5’-AGGAAACAGCTATGACCATG), and PCR products were sequenced in both directions on an ABI 3700 capillary sequencer available at Eton Biosciences Inc (San Diego, CA, USA).

Sanger reads were de-novo assembled in Geneious Prime^®^ 2021.1.1. Plasmid DNA sequences were removed from reads and at least 25 bp from each end were trimmed. Consensus contigs were manually edited as needed to resolve conflicts between forward and reverse reads. Consensus contigs were aligned in Mesquite v2.75 [52] using MUSCLE v3.8.425 [53] with the number of maximum iterations set at 8.

The alignment was trimmed to 725 bp to remove primer annealing sites and misaligned ends. The alignment was filtered to identify and remove potential nuclear mitochondrial DNA and chimeric sequences (NUMTS). In total, 127 sequences were removed based on the following criteria [54]: sequences containing multistate characters, indels, single-tons, and stop codons. The pseudogene ‘MEAM2′ [55,56] was used to root the tree.

### 2.2. Variant Calling and Population Differentiation

In total, 503 sequences representing the 3′-end of the mtCOI gene were aligned and evaluated for informative single nucleotide polymorphisms. The Arizona B-prototype (AzB) sequence (Accession no. AY057123) was included for comparison. Variants were called using the ‘Find variations/SNPs’ option implemented in Geneious prime v2019.1.3 (Biomatters Ltd., Auckland, New Zealand) with customized parameters at minimum coverage of 7 and a minimum of 50% variant frequency.

To determine whether the SNP-defined haplotypes belonged to a single or multiple populations, an exact test of population differentiation was carried out in Genepop v4.7 [57] with Markov chain parameters, as follows: dememorization number of 1 × 105, 1000 batches, and 1 × 105 iterations per batch. An SNP matrix was entered manually in a text editor, where rows consisted of all the SNPs found across all SNP-defined haplotypes, and the profile groups or populations were listed in columns with SNPs locations. The matrix was converted to the ‘genepop’ format by using the snp2gp function from the package Diversity in R [58]. Significance values at the 0.05 confidence level were corrected using the standard Bonferroni correction.

### 2.3. Phylogeography of Haplotypes

The best-fit model of evolution was determined using jmodeltest software v2.1.7 [59] based on the corrected Akaike information criterion (AICc). The Hasegawa-Kishino-Yano model of evolution (HKY) [60] was identified as the optimal model. Bayesian phylogenetic inference (BI) was carried out with MrBayes v 3.2.7 [61], with four independent computations, consisting of four Markov chains each run for 3 × 10^7^ generations. Trees were sampled every 1000 generations and log-likelihood scores were visualized using Tracer v 1.6 [62] to verify the convergence of the chain and to determine the optimal “burn-in” fraction. Runs with effective sample sizes (ESSs) of ≥200 were combined using the LogCombiner software v1.8.4. The optimal burn-in was set at 1 × 10^8^ generations. The 70% majority-rule consensus tree was obtained with TreeAnnotator v1.8.4 and visualized using FigTree v1.4.2 (http://tree.bio.ed.ac.uk/software/figtree/, accessed on 15 January 2020).

### 2.4. Network of Haplotypes

The aligned matrix, which contained 315 unique mtCOI-3′ haplotypes representing all SNP profiles, was used to build a median-joining network of haplotypes using Network v10.1.0.0 [63]. Sequences were formatted in “nexus sequential” format, and the alignment was imported to the Network v 5.0.0.3 software (Fluxus Technology Ltd., Suffolk, UK), available at http://www.fluxus-engineering.com/sharenet.htm, accessed on 8 December 2019. Each site of the alignment was weighted uniformly with the epsilon (η) value set to zero.

### 2.5. Eco-Geographic Distribution of Haplotypes

The geographic coordinates associated with the aligned sequences were downloaded from NCBI using the Biopython module “Entrez” [64]. When NCBI records lacked geographic coordinates but provided well-documented collection sites, the coordinates were identified manually using Google Maps (www.maps.google.com, accessed on January 2021). The ‘occurrence records’ of the B mitotype were drawn with the biological records tool plugin, available in QGIS 3.8.1 [65], and overlaid to the world map Köppen-Geiger climate classification [48,66] climatological layer, and the polygon that defines the Irano-Turanian floristic region [67].

## 3. Results

### 3.1. SNP-Defined Haplotypes of the B Mitotype

**Figure 1:**
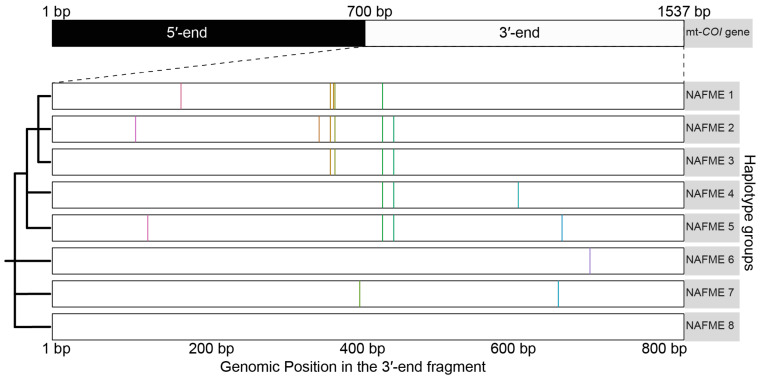
Schematic representation of the ‘signature SNPs’, or those that differentiated the eight unique haplotypes groups of the *Bemisia tabaci* B mitotype. Color bars indicate the locations of signature SNPs identified in the 3′-end of the COI gene.

The 509 sequences were differentiated as eight haplotypes, designated NAFME 1–8 (Figure 1). Haplotype NAFME 6 had one SNP that resulted in a predicted amino acid change, and the remaining 12 SNPs across the NAFME haplotypes were identified as silent mutations (Table 1). The NAFME 1 haplotype sequence had five silent SNPs, four transitions, and one transversion. The NAFME 2 haplotype harbored six silent polymorphisms comprising five transitions and one transversion. The NAFME 3 haplotype harbored three silent transitions and one transversion. The NAFME 4, 5, and 7 showed silent transitions only. The NAFME 1–3 shared three common SNPs, whereas NAFME 4 and 5 shared either one to two SNPs (Figure 1). Of the eight NAFME haplotypes, two, the NAFME 6 and 8, occurred outside of their presumed native niche range. By comparison, as a result of multiple introductions, NAFME 8 (AzB prototype) has a cosmopolitan distribution, and NAFME 6, the only other known invasive haplotype, also occurs in China and India. All of the other haplotypes were found exclusively within their predicted center of origin in the NAFME region.

### 3.2. Phylogenetic Analysis

**Figure 2:**
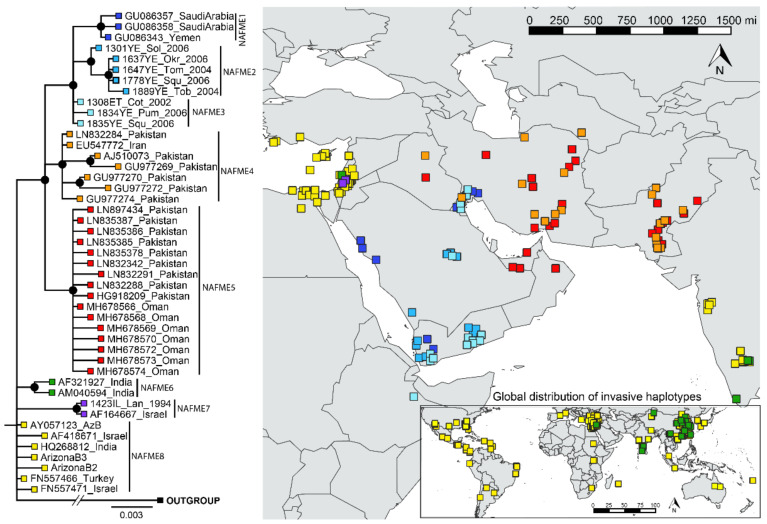
Phylogeography of all known B mitotype haplotypes of *Bemisia tabaci*. The phylogenetic breaks coincide with the SNPs classification of haplotypes given in Table 1.

The phylogenetic breaks of all B mitotype haplotypes, except NAFME 3 and 8, were well-supported (100% posterior probability), and were concordant with the SNP-mediated classification of haplotypes NAFME 1–8 (Figure 2). Despite NAFME 3 and 8 forming polytomies, SNPs were found fixed in all sequences, which allowed their classification into two different haplotypes (Figure 1). The NAFME 1–3 clustered in a monophyletic clade. The NAFME 4 and 5 are sisters to the NAFME 1–3 clades, and together form a well-supported monophyly. The NAFME 6–8 clades showed no evidence of sistership to NAFME 1–5, and instead formed separate clades with unresolved sister relationships. Support for the respective predicted phylogeographic distributions was robust (Figure 2). The NAFME 1–3 haplotypes were affiliated with the Arabian Peninsula and Ethiopia (east coastal Africa), and NAFME 4 and 5 shared one or two SNPs with Arabian Peninsula haplotypes but had a distribution that was restricted to Iran, Iraq, Pakistan, and Turkmenistan (Table 1). The NAFME 5 was also identified as extant in the United Arab Emirates and Oman, which are located in the Arabian Peninsula. These records indicate the possible introduction of the NAFME 5 haplotype from the semi-arid lands of Iran, where they appear to be native to various desert locales in the Arabian Peninsula. This prediction is supported by the unprecedented massive outbreaks reported in okra fields in Oman, where some of the whitefly samples studied herein were collected. The NAFME 6 haplotype was detected in its predicted native range in Israel, as well as in East Asia and India, where it is known to represent a recent introduction [23,25]. The NAFME 7 haplotype was found only in Israel, whereas haplotype NAFME 8 was found both in the Mediterranean region of the Middle East and the southern deserts of Israel, from where it is predicted to have been introduced to non-native micro-climate niches in tropical and subtropical agroecosystems, nearly worldwide (Figure 2).

### 3.3. Network Analysis and Population Differentiation

**Figure 3:**
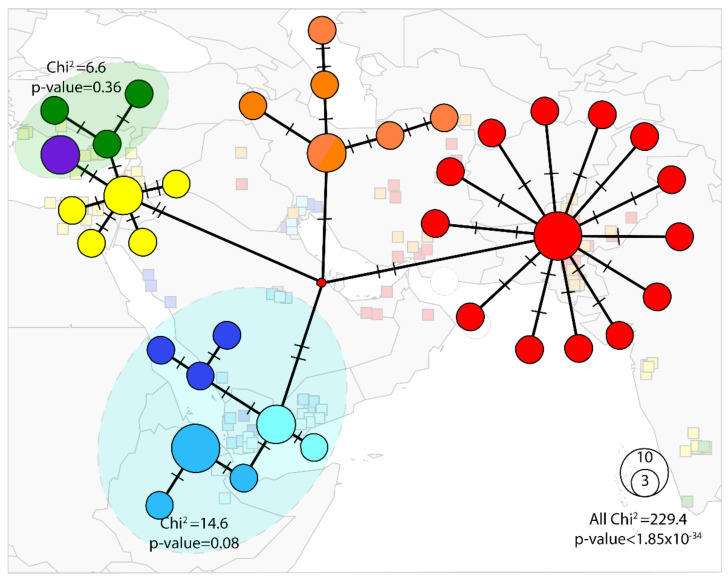
Median joining network of the *Bemisia tabaci* B mitotype (ε = 0). The blue shading indicates POP1, the single population comprising haplotypes NAFME 1–3. The orange-colored circles represent POP2 constituted by NAFME 4. The red circles indicate POP3 formed by haplotype NAFME 5. Green circles demarcate haplotype NAFME 6, the purple circle indicates haplotype NAFME 7, which together form population POP4. Yellow circles denote haplotype NAFME 8, which alone constitute population POP5. Relative abundance is represented by the size of each circle. Black lines show the relatedness between haplotypes, and lines shown on branches indicate the number of mutations.

The phylogeographic distributions of the haplotype were concordant with the network analyses (Figure 3) except for NAFME 6–8, which showed evidence of having an interconnected network, albeit unresolved phylogenetic relationships. The NAFME 1–3 haplotypes also showed an interconnected network, but their phylogenetic relationship was well-supported. By comparison, although the geographical distribution of haplotypes NAFME 4 and 5 overlapped and shared selected SNPs, they were affiliated with independent sub-networks, a relationship that was also supported by the phylogenetic tree structure. Finally, haplotype 5 showed a star-like structure, i.e., a predominant haplotype being surrounded by less abundant haplotypes that harbor few mutations, a pattern indicative of a previous regional demographic expansion of NAFME 5.

An ‘exact test’ of population differentiation showed that the eight haplotypes consisted of five well-differentiated genetic populations (*p* < 0.05), hereon POP1–5. Haplotypes NAFME 1–3 comprised a single population (*p* = 0.08), the POP1, representing the warm deserts of the Arabian Peninsula and Ethiopia. By comparison, the NAFME 4 and 5 haplotypes were distinct populations, POP2 and POP3, respectively, which distributed across warm and cold semi-arid regions of the northern ranges of the Middle East, respectively, within the Irano-Turanian floristic region, characterized by extreme endemism of its many life forms [67]. Because NAFME 5 is known to be extant in the deserts of Oman and UAE, it either is endemic in all locales, or may possibly represent a recent introduction. Finally, haplotypes 6 and 7 represented one population, POP4, which co-occurs in the western Mediterranean region of the Middle East and western coast of Turkey (Figure 3), whereas the NAFME 8, which constitutes one population, POP5, spans the Mediterranean and desert niches of the Middle East regions.

### 3.4. Eco-Geographic Distribution of Haplotypes

**Figure 4:**
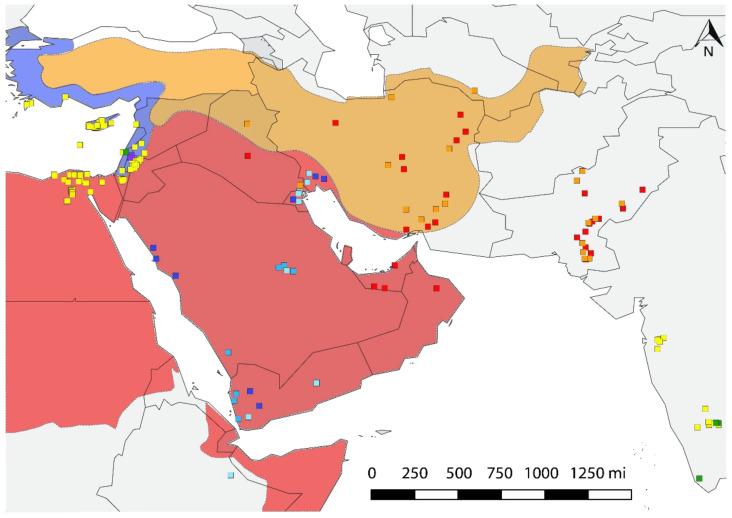
Distribution of haplotypes in ecological macro niches. Red area represents the hot desert climate (BWh) of the Arabian Peninsula and Northeast Africa. Orange shows the Irano-Turanian floristic region with semi-arid ecosystems (BSh, BSk). Blue indicates the eastern Mediterranean niches (Csa). NAFME 1–3 inhabit desert habitats, NAFME 4 and 5 are native to the semi-arid regions, while NAFME 6 and 7 inhabit only those locales in the Middle East with a Mediterranean-like climate, and NAFME 8 occupies both locales in the western Mediterranean-deserts of Israel and desert habitats in Egypt where irrigated agriculture has long been practiced, e.g., the Red Sea coast.

The hypothetical center of the origin of *B. tabaci* in the Middle East represents three eco-geographical units based on climate and endemism indicators that are consistent with the extant Mediterranean, desert, and the Irano-Turanian regions (Figure 4). The Mediterranean region encompasses Cyprus, Lebanon, northern Israel, and the coastal lands of Turkey and Syria. The desert region consists of Bahrain, Kuwait, southern Israel, Oman, Saudi Arabia, United Arab Emirates, Yemen, and portions of Iran, Iraq, Jordan, and Syria. The Irano-Turanian is a region of high endemism, spanning much of Iran and Turkey and northern Iraq and Syria. The NAFME 1–3 is predicted to be endemic to the Middle Eastern deserts, whereas the range of NAFME 4 and 5 haplotype endemism spans the Irano-Turanian region, haplotypes NAFME 6 and 7 are native to the Mediterranean region occurring in the Middle East, and the NAFME 8 haplotype originates in adjacent overlapping Mediterranean and desert niches (Figure 4). Thus, the NAFME 6 and 8 haplotypes are the only known B mitotypes known to have established outside of their predicted zone of endemism within northeastern coastal Africa (here, represented by Ethiopia collections) and the Middle East. The NAFME 4 and 5 were also identified among collections from Oman, southern Pakistan, and the United Arab Emirates.

## 4. Discussion

The mtCOI gene, particularly the 3′-end region [50], has been demonstrated to be highly reliable for pinpointing in close proximity the extant phylogeographic origins of the *B. tabaci* cryptic species (see references in [2]). Analogously, it has been widely favored for the phylogeographical differentiation of many eukaryotic organisms and so is frequently favored as a marker for resolving intraspecific relationships [42]. In this study, the mtCOI B mitotype-signature SNPs have corroborated the reliability of the mtCOI as a molecular marker and expanded its utility for differentiating haplotypes at a finer scale. Based on SNPs occurrence uniquely and/or shared in common, eight phylogeographically unique haplotypes of the B-mitotype of *B. tabaci* were differentiated in the NAFME region.

The GenBank database houses an important archive of mtCOI sequences [68], which increased exponentially after initial reports that demonstrated the mtCOI robustness to resolve phylogeographic clades [1,46] and for differentiating putative cryptic species [69]. However, subsequent studies have revealed the inadvertent amplification of whitefly mtCOI pseudogenes [56,70] that along with potential chimeras and singletons often result from PCR amplification using low fidelity polymerases and low-coverage sequences [71]. Singletons are problematic in phylogenetic studies at the microevolutionary level because as much as one character state that appears most parsimonious could lead to an erroneous phylogenetic conclusion [72]. Thus, stringent curation was implemented when recovering the well-defined phylogenetic breaks for haplotypes of the B mitotype identified in this study.

Phylo- and eco-geographic breaks are correlated and support the existence of several NAFME haplotypes, of which only NAFME 6 and 8 have been introduced to tropics and subtropics of the world in the last three decades [6]. Another haplotype that could feasibly upsurge is NAFME 5, which has shown genetic signatures of genetic and demographic expansion. Notably, the NAFME 5 has been identified in the easternmost desert of the Arabian Peninsula, in Oman and the United Arab Emirates, a predicted niche suitable primarily for native NAFME 1–3. In Oman, NAFME 5 has been identified recently in heavily infested okra fields and previously unreported begomovirus outbreaks (I. Haq, personal communication). Analogous scenarios have resulted in the rapid displacement of the native mitotypes [19,73], which may explain the failure to find native NAFME 1–3 in these locales. An alternative explanation for the occurrence of NAFME 5 at the southern edge of the Arabian Peninsula is their potential adaptation to this particular micro-niche, which is influenced by Indian Ocean Monsoons that favor seasonal flourishes of vegetation casts. More rigorous sampling of the diverse natural and agro-ecosystems in the Arabian Peninsula is expected to provide additional insights into the distribution of *B. tabaci* haplotypes in the region.

Unexpectedly, NAFME 4 and 5 haplotypes were also found in southern desert Pakistan, a country where Asia II mitotypes predominate [50,74] and B-relatives were identified using the mtCOI marker only by 2007 [74,75]. What might look like a plausible introduction of NAFME 4 and 5 either by a migration corridor through Afghanistan or via anthropogenic transport of goods could represent traces of the drastic habitat fragmentation history of the Irano-Turanian zone driven by climatic oscillations in the last quaternary glacial-interglacial cycles [76] that ended c.20 Kya [77] and the gradual aridification and cooling of the region over the last 20 Ma [78,79]. Under these circumstances, the NAFME 4 and 5 might have specialized in native flora of no agricultural importance in Pakistan, thence it might had been overlooked by whitefly surveys there before 2007.

In Iran, NAFME 4 or 5 have been reported as important pests and plant virus vectors [80], and recently, the propensity for high fecundity of certain haplotypes became a public health threat [81] in that urban outbreaks lead to human respiratory complications [81,82,83,84]. Clearly, tracking these ‘high-risk’ *B. tabaci* haplotypes has become an urgent short-term need to abate the gradual expansion of these populations across neighboring countries. An alternative explanation to the unprecedented urban outbreaks in large cities in Iran could be that invasive NAFME 8, the resilient haplotype, has only recently dispersed to northern Iran, where it has outcompeted NAFME 4 and 5; however, a larger sample size is needed to corroborate this interpretation.

The most parsimonious explanation for the intra-mitotype phylogenetic breaks and discontinuous genetic networks with a strong geographic orientation among haplotypes of the B mitotype is the existence of long-term geographic (e.g., of the zoogeographic type) barriers to gene flow [42]. Here, we present biogeographic and genetic evidence that climate zones and high endemism hotspots might have shaped the evolution of the intra-mitotype diversity in the *B. tabaci* B mitotype by impeding gene flow among these populations for a long time so that clear differences have also accumulated in their nuclear genome (Paredes-Montero, unpublished data). In addition, the ecology of whitefly species in desert habitats might also explain the diversification of these haplotypes in the Middle East in the context of isolation by distance principles. In such habitats, host species are mainly available along flowing rivers and washes and contract or expand seasonally, which directly influence whitefly dispersal. These observations do not preclude the possibility that some haplotypes might have occupied ranges free of firm impediments to gene flow, such as biogeographic transition zones in Kuwait, south Iraq, and Syria, which could potentially lead to hybridization events that give rise to resilient NAFME hybrids capable of withstanding broader ranges of climatic conditions than parental haplotypes. The nuclear genomes of representative populations are currently being studied to test this hypothesis.

The delimitation and cohesiveness of NAFME haplotype assemblages within well-delineated biogeographical regions in the Middle East reinforce the ‘out-of-Africa’ migration hypothesis [85] of the *B. tabaci* evolution. Based on the proposed scenario, NAFME founder populations from the Arabian Peninsula (extant) spread into Eurasia during the late Oligocene when the Arabian and Eurasian plates collided ~25 Mya. The uplifting of mountain ranges and plateaus between 15 to 5 Mya was favorable for allopatric diversification of *B. tabaci*, while populations in west Eurasia (Mediterranean) became isolated by the Taurus and Alborz Mountain ranges and the Anatolian plate, and eastern and western Eurasian populations were from counterparts in the Arabian Peninsula by the Zagros Mountain range. These geological events match previous divergence estimates that place the radiation of *B. tabaci* between 19.52 to 3.43 Ma [86,87,88]. The east Eurasian founder population may have evolved into the extant Q mitotype or MED cryptic species, an event that likely occurred before the diversification of NAFME 1–8 haplotypes, a hypothesis that is supported by a 6% nucleotide divergence between the Q and B mtCOI sequences [2]. The latter estimate is greater than the initial nucleotide divergence estimates among NAFME haplotypes, of approximately 3% (albeit small sample size) [46] or more [1,20]. Therefore, gene flow among NAFME haplotypes continued to occur until the uplift of the Zagros Mountain range that isolated NAFME 4 and 5 from the rest in the Arabian Peninsula. Other factors might have shaped the evolution of haplotypes that live in sympatry, such as host specialization [89,90], endosymbiont assemblages [91], climate gradients [92], sky islands, and/or micro-geological events [93] within Middle Eastern plateaus.

**Figure 5:**
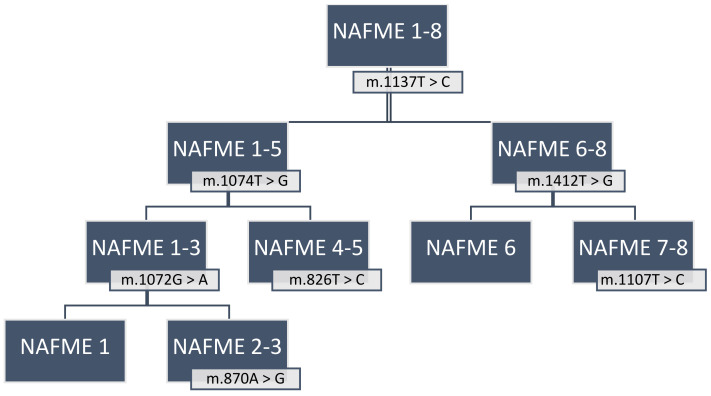
Flowchart to identify haplotypes of the *Bemisia tabaci* B mitotype using the newly characterized signature SNPs. NAFME (North Africa—Middle East) 1–8 are biogeographic designations of the *B*. *tabaci* B haplotype groups delineated in this study.

Application of the B-haplotype-specific SNPs for the identification of NAFME 1–8 (Figure 5) by PCR amplification and DNA sequencing of the 3′-mtCOI fragment requires the use of a high-fidelity DNA polymerase, preferably with 3′ to 5′ exonuclease activity [71,94]. To avoid chimeras, pseudogenes, and singletons, cloning and bi-directional sequencing of amplicons are essential, and cloning of at least three amplicons per sample is advisable.

## Figures and Tables

**Table 1:** Summary and description of signature single nucleotide polymorphisms (SNPs) used for differentiation of haplotypes of the B mitotype of *Bemisia tabaci*. The North Africa-Middle East (NAFME) region is the extant origin of haplotypes used in this study, based on collection sites.

NAFME Haplotypes	Country	SNP	Polymorphic Type	Variant Frequency	Variant Coverage	Variant *p*-Value *
NAFME 1	Iran, Kuwait, Saudi Arabia, Yemen	m.870A > G	transition	66.70%	7	1.50 × 10^−7^
		m.1074T > G	transversion	100.00%	7	1.00 × 10^−12^
		m.1068T > C	transition	100.00%	7	1.00 × 10^−12^
		m.1137T > C	transition	100.00%	7	1.00 × 10^−12^
		m.1072G > A	transition	100.00%	7	1.00 × 10^−12^
NAFME 2	Saudi Arabia, Yemen	m.1074T > G	transversion	100.00%	10	1.00 × 10^−18^
		m.810T > C	transition	88.90%	10	8.90 × 10^−12^
		m.1068T > C	transition	100.00%	10	1.00 × 10^−18^
		m.1137T > C	transition	100.00%	10	1.00 × 10^−18^
		m.1152T > C	transition	100.00%	10	1.00 × 10^−18^
		m.1053G > A	transition	100.00%	10	1.00 × 10^−18^
NAFME 3	Ethiopia, Iran, Kuwait, Saudi Arabia, Yemen	m.1074T > G	transversion	100.00%	14	1.00 × 10^−26^
		m.1068T > C	transition	100.00%	14	1.00 × 10^−26^
		m.1137T > C	transition	84.60%	14	7.70 × 10^−21^
		m.1152T > C	transition	61.50%	14	1.20 × 10^−13^
NAFME 4	Iran, Iraq, Pakistan, Turkmenistan	m.1137T > C	transition	100.00%	16	1.00 × 10^−30^
		m.1152T > C	transition	100.00%	16	1.00 × 10^−30^
		m.1317T > C	transition	100.00%	16	1.00 × 10^−30^
NAFME 5	Iran, Iraq, Oman, Pakistan, United Arab Emirates	m.1375C > T	transition	100.00%	33	1.00 × 10^−64^
		m.826T > C	transition	100.00%	33	1.00 × 10^−64^
		m.1137T > C	transition	93.80%	33	4.90 × 10^−58^
		m.1152T > C	transition	100.00%	33	1.00 × 10^−64^
NAFME 6	China, India, Israel	m.1412T > G	transversion	100.00%	7	1.00 × 10^−14^
NAFME 7	Israel	m.1383T > C	transition	100.00%	7	0.0001
		m.1107T > C	transition	100.00%	7	0.0001
NAFME 8 ^¥^ (AzB)	Argentina, Brazil, China, Colombia, Costa Rica, Cyprus, Ecuador, Egypt, Guatemala, India, Israel, Malaysia, Saudi Arabia, South Korea, Trinidad and Tobago, Turkey, USA, and others ^α^					

* Probability of sequencing error resulting in bases with at least the given sum of qualities. ^¥^ Haplotype included as the reference sequence for SNP searches. ^α^ Representative countries where NAFME 8 has been introduced.

## Data Availability

The DNA sequences obtained through with this research have been deposited in the NIH-NCBI-GenBank database. Accession numbers are available in the Supplementary Table S1.

## Supplementary Materials

The following are available online at www.mdpi.com/article/10.3390/biology10101048/s1.
